# Multi-Omics Profiling Specifies Involvement of Alternative Ribosomal Proteins in Response to Zinc Limitation in *Mycobacterium smegmatis*

**DOI:** 10.3389/fmicb.2022.811774

**Published:** 2022-02-10

**Authors:** Allexa Dow, Andrew Burger, Endrei Marcantonio, Sladjana Prisic

**Affiliations:** ^1^School of Life Sciences, University of Hawai‘i at Mānoa, Honolulu, HI, United States; ^2^School of Ocean and Earth Science and Technology, University of Hawai‘i at Mānoa, Honolulu, HI, United States

**Keywords:** mycobacteria, ribosomal proteins, zinc, *Mycobacterium smegmatis*, multi-omics, transcriptome, proteome, oxidative stress response

## Abstract

Zinc ion (Zn^2+^) is an essential micronutrient and a potent antioxidant. However, Zn^2+^ is often limited in the environment. Upon Zn^2+^ limitation, *Mycolicibacterium* (basonym: *Mycobacterium*) *smegmatis (Msm)* undergoes a morphogenesis, which relies on alternative ribosomal proteins (AltRPs); i.e., Zn^2+^-independent paralogues of Zn^2+^-dependent ribosomal proteins. However, the underlying physiological changes triggered by Zn^2+^ limitation and how AltRPs contribute to these changes were not known. In this study, we expand the knowledge of mechanisms utilized by *Msm* to endure Zn^2+^ limitation, by comparing the transcriptomes and proteomes of Zn^2+^-limited and Zn^2+^-replete *Msm*. We further compare, corroborate and contrast our results to those reported for the pathogenic mycobacterium, *M. tuberculosis*, which highlighted conservation of the upregulated oxidative stress response when Zn^2+^ is limited in both mycobacteria. By comparing the multi-omics analysis of a knockout mutant lacking AltRPs (Δ*altRP*) to the *Msm* wild type strain, we specify the involvement of AltRPs in the response to Zn^2+^ limitation. Our results show that AltRP expression in *Msm* does not affect the conserved oxidative stress response during Zn^2+^ limitation observed in mycobacteria, but AltRPs do significantly impact expression patterns of numerous genes that may be involved in morphogenesis or other adaptive responses. We conclude that AltRPs are not only important as functional replacements for their Zn^2+^-dependent paralogues; they are also involved in the transcriptomic response to the Zn^2+^-limited environment.

## Introduction

Zinc ion (Zn^2+^) is an important micronutrient, but many bacteria encounter Zn^2+^-limited environments (Corbin et al., 2008; Alloway, 2009). Acting as a cofactor to many proteins (Andreini et al., 2006) and as a potent antioxidant (Powell, 2000), the presence or absence of Zn^2+^ can affect bacterial physiology (Lim et al., 2013; Dow and Prisic, 2018; Velasco et al., 2018). For example, in the human pathogen *Mycobacterium tuberculosis* (*Mtb*), Zn^2+^-limitation induces vast changes in expression patterns of the transcriptome and proteome, correlating with increased resistance to oxidative stress and increased virulence of Zn^2+^-limited *Mtb* (Dow et al., 2021). A marked feature of Zn^2+^-limited *Mtb* was upregulation of the oxidative stress response, evidenced by increased expression of key antioxidant enzymes and genes involved in DNA repair (Dow et al., 2021).

The adaptations to Zn^2+^ limitation could be driven by the fact that *Mtb* transits through a Zn^2+^-depleted niche (i.e., caseum and sputum) during active tuberculosis (Dow et al., 2021). However, environmental bacteria such as *Mycolicibacterium* (basonym: *Mycobacterium*) *smegmatis* (*Msm*) may also encounter Zn^2+^ limitation, and so these adaptations could span beyond the context of the infection. Determining if these adaptations to Zn^2+^ limitation are unique to tuberculosis for *Mtb* or conserved across mycobacteria will indicate the importance of this adaptive response in (myco)bacteria.

We anticipate vast and unique changes in gene and protein expression patterns in response to Zn^2+^ limitation in *Msm* compared to *Mtb*, especially considering that the previously observed morphological changes (elongated cells with condensed DNA) in Zn^2+^-limited *Msm* do not occur in *Mtb* (Dow and Prisic, 2018; Dow et al., 2021). Moreover, the Zn^2+^-dependent morphogenesis in *Msm* relies on expression of Zn^2+^-independent alternative ribosomal proteins (AltRPs) (Dow and Prisic, 2018). The conserved mycobacterial *altRP* operon contains four genes encoding AltRPs that replace their primary homologues in the ribosomes of Zn^2+^-limited mycobacteria (Dow and Prisic, 2018; Li et al., 2018; Tobiasson et al., 2019). Although implicated in shaping mycobacterial physiology, the exact role of AltRPs is unclear (Dow and Prisic, 2018). Because AltRPs are incorporated into functional ribosomes that engage in active translation (Tobiasson et al., 2019), it has been suggested that AltRP-containing ribosomes may selectively translate certain mRNAs (Chen et al., 2020). Therefore, the “failure” of a mutant lacking the *altRP* operon to undergo the Zn^2+^-dependent morphogenesis could be due to a checkpoint “failure” in translation, since this strain can only build ribosomes with Zn^2+^-dependent (or “primary,” Prim) paralogs. Tracking gene expression patterns (i.e., transcript/protein pairs) in the wild type and a mutant lacking AltRPs (Δ*altRP*) during Zn^2+^-limitation will further enlighten the role of AltRPs in mycobacterial physiology.

In this study, we employ a multi-omics analysis of Zn^2+^-limited *Msm* and compare the results to those described for *Mtb* to determine whether the link between Zn^2+^-limitation and specific transcriptional responses, particularly regulation of the oxidative stress response, is conserved in mycobacteria. We further describe the effects of Zn^2+^-limitation in *Msm* beyond those that are shared with *Mtb*. Given the role of AltRPs in the Zn^2+^-dependent morphogenesis in *Msm*, we compare the transcriptome and proteome of the Δ*altRP* mutant and the wild type, to inform how AltRP expression contributes to the adaptation to Zn^2+^-limitation in mycobacteria. Together, this multi-omics analysis of *Msm* grown in Zn^2+^-replete media (ZRM) and Zn^2+^-limited media (ZLM), along with the Δ*altRP* mutant and the *in trans* complement strain (Δ*altRP/c*), also grown in ZLM, provides molecular details on how Zn^2+^ and AltRP expression contributes to mycobacterial physiology.

## Materials and Methods

### Media, Strains and Bacterial Growth

All chemicals were purchased from Thermo Fisher Scientific™ or VWR™, unless otherwise noted. Middlebrook 7H9 (Difco) broth supplemented with ADC-T (0.5% albumin, 0.2% glucose, 0.085% NaCl, 0.05% Tween 80) was used to maintain *Msm* strains. Preparation of chemically defined Sauton’s medium (0.05% KH_2_PO_4_, 0.05% MgSO_4_7H_2_O, 0.2% citric acid, 0.005% ferric ammonium citrate, 6% glycerol, 0.4% asparagine, 0.05% Tween 80, pH 7.4) was done with care to avoid Zn^2+^ contamination from glassware as previously described (Dow et al., 2021). Omitting ZnSO_4_ in Sauton’s Zn^2+^-limited medium (ZLM) yields a final Zn^2+^ concentration around 100 nM, detected by ICP-MS (Dow et al., 2021). For Zn^2+^-replete medium (ZRM), ZnSO_4_ was added to ZLM at 6 μM concentration.

Wild type *Msm* (mc^2^ 155), the deletion mutant of the *altRP* operon (Δ*altRP*) and its complement strain (Δ*altRP*/c) were constructed for a previously published study (Dow and Prisic, 2018). Zn^2+^-limitation was achieved through growth in ZLM to stationary phase as previously described (Dow and Prisic, 2018), at which time cells were harvested for analysis (i.e., 72 h after inoculation). Zn^2+^-replete cells (i.e., wild type grown in ZRM) were harvested at the same time as the Zn^2+^-limited cells.

### Isolation of RNA and Proteins

From 50 mL cultures, 25 mL of each culture was transferred to 50 mL conical tubes and cells were pelleted with centrifugation at 3,000 × *g* for 10 mins at 4°C. The supernatant was discarded, and cell pellets were resuspended in 1 mL TRIzol™ reagent (Invitrogen™, Cat. #: 15596026) and transferred to a 2 mL screw-cap tube containing 200 μL of 0.1 mm zirconia beads (BioSpec, Cat. #: 11079101z). Bacterial cell pellets in TRIzol™ were lysed via beating 3 times for 45 seconds at 7000 rpm in a MagNA Lyser (Roche) with cooling on ice for 3 mins between cycles. Screw cap tubes were spun at 12,000 × *g* for 5 mins at 4°C and the supernatant was transferred to new 1.5 mL tubes. RNA and proteins were extracted from TRIzol™ supernatants concurrently, following the manufacturer’s protocol. Proteins were resuspended in 9.5 M urea and 2% CHAPS buffer, pH 9.1 and stored at −80°C.

Precipitated RNA from the aqueous TRIzol™ fraction was resuspended in nuclease free water and purified using High Pure RNA Isolation kit (Roche, Cat. #: 11828665001) with omission of the on-column DNase digest step. Purified RNA was quantified using the ratio of absorbance at 260 and 280 nm using a Nanodrop™ (Thermo Fisher Scientific™) spectrophotometer and digested in solution twice with TURBO™ DNase (Invitrogen™, Cat. #: AM2239) following the manufacturer’s directions. DNase-digested RNA was re-purified using the same kit.

### High-Throughput RNA Sequencing and Differential Expression Analysis

Ribosomal RNA was depleted from the samples using Ribo-Zero plus rRNA depletion kit (Illumina^®^, Cat. #:20037135) before being sequenced on an Illumina^®^ MiSeq platform for generation of 150 base pair paired-end reads. RNA-seq was performed by GENEWIZ^®^ (South Plainfield, NJ 07080). Analysis of raw read data was achieved following the pipeline: fastQC (Andrews, 2010) of raw reads in .fastq format, trimmomatic (Bolger et al., 2014), fastQC of trimmed reads, Bowtie 2 (Langmead and Salzberg, 2012) alignment of trimmed files and featureCounts (Liao et al., 2014) to define tagwise abundances for coding and non-coding RNAs from BAM files. Reference genome files were obtained from NCBI using the *M. smegmatis* mc^2^ 155 reference genome assembly (ASM1500v1.39). We manually curated certain gene annotations in the tables when the gene was mentioned in the text but did not have an associated gene annotation in the reference file. The gene names were inferred from orthologues with gene annotations in *Mtb*, or from gene names reported in the literature for *Msm*. Homologues between *Msm* and *Mtb* were obtained from Mycobrowser, Release 3 (Kapopoulou et al., 2011). Data analysis was performed through the command line using the high-performance computing cyberinfrastructure from the University of Hawaii Information Technology Services.

Genes with tagwise abundances of less than 10 counts in all strains and conditions were considered lowly expressed and excluded from the differential expression analysis. The tagwise abundances were normalized using trimmed means of *M*-values (TMM) method in edgeR to obtain logCPM values for each feature (Robinson et al., 2010) and the quantile-adjusted conditional maximum likelihood (qCML) linear modeling approach of normalized gene abundances was used to determine the common dispersion using limma (Ritchie et al., 2015). Differential gene expression of the linearized model was determined using an “exact-like test” that utilizes a negative binomial distribution model and Benjamini-Hochberg false discovery rate (FDR) approximation to control for the family wise error rate (Ritchie et al., 2015). Significantly differentially expressed genes (DE genes) were defined as having an absolute fold change (absFC) greater than 2 and an FDR (adjusted *P*-value) less than 5% (FDR < 0.05, absFC > 2). A log_2_-fold change (lfc) threshold of 1 was applied using *treat* function in limma to ensure the false discovery rate (FDR) is controlled using the Benjamini-Hochberg procedure for multiple testing correction while only considering genes with changes in expression levels above the threshold (absFC > 2) (Ritchie et al., 2015). RNA sequence analysis using the edgeR-limma workflow was based on the previously published script (Law et al., 2018) and was performed using R version 4.1.0 (R Core Team, 2019).

Enrichment analysis for gene ontology (GO) terms from DE genes in was achieved using DAVID with a *p*-Value < 0.05 used to define significantly enriched GO terms unless otherwise stated (Ashburner et al., 2000; Huang et al., 2009a,b). The circle plots were created using the R package GOplot version 1.0.2 (Walter et al., 2015). The color of the bars in the inner circle of the circle plots and the bar graphs is determined by the z-score which indicates whether the given biological process is more likely to be decreased or increased in the dataset and is given as follows:


$$z\,s\,c\,o\,r\,e=\frac{\left(u\,p-d\,o\,w\,n\right)}{\sqrt{c\,o\,u\,n\,t}}$$

Whereas *up* and *down* are the number of DE genes that are upregulated or downregulated, respectively in the comparison and *count* is the total number of genes in the GO term. Superimposition of DE genes onto the global metabolic network and carbon metabolism pathways was achieved using KEGG (Kanehisa, 2000; Kanehisa et al., 2019).

### Protein Profiling Using Label-Free Quantitative Mass Spectrometry and Differential Expression Analysis

Total cellular protein was isolated from the organic fraction from TRIzol™ extractions above, and proteins were purified as previously reported (Prisic et al., 2015). Protein concentration of solubilized pellets was determined with the DC assay (Bio-Rad). Protein (100 μg) was digested using filter-aided sample preparation (FASP) method (Wiśniewski and Rakus, 2014) and digested peptides were resuspended in 5% acetonitrile/5% formic acid and analyzed by UC Davis Genome Center – Proteomics Core by LC-MS/MS on a Q Exactive™ Plus Orbitrap Mass spectrometer in conjunction with Proxeon Easy-nLC II HPLC (Thermo Fisher Scientific) and Proxeon nanospray source following the Core’s standard protocol. Buffer A was 0.2% formic acid in water and buffer B was 0.2% formic acid in acetonitrile. The following method was used: flow rate was 2 μL/min, 10 min buffer A, 90 min 5-35% gradient buffer B, 5 min 95% buffer B, 5 min 5% buffer B. Mass spectra in .raw format was converted to .mzXML format using MSConvert from ProteoWizard (ProteoWizard 3.0.19317.0ef6e44d0). Tandem mass spectra were extracted, and MS/MS analyzed with X! Tandem for peptide identification using the following search parameters: cysteine alkylation – iodoacetamide; digestion – trypsin; fixed modifications – carbamidomethylation (C); variable modifications – oxidation (M), deamidation (N,Q), phosphorylation (S,T,Y), and acetylation (K); precursor mass tolerance – 20ppm and fragment mass tolerance – 10ppm (The GPM, thegpm.org; version X! Tandem Alanine (2017.2.1.4)) (Craig and Beavis, 2004). Peptides were searched in X! Tandem for protein identification using a custom .fasta file of protein sequences from *M. smegmatis* mc^2^ 155 reference genome assembly (ASM1500v1.39), obtained from NCBI. The reverse sequences of all proteins were included in the .fasta file as decoy sequences. It has been demonstrated that multidimensional proteomic datasets from label-free quantitation experiments have a mean-dispersion relationship that can be modeled in edgeR, and as such we used the same edgeR-limma workflow and cutoff values to define significance as applied to the RNA-seq dataset for differential expression analysis of the proteome using spectral counts from X! Tandem as tagwise abundances (Branson and Freitas, 2016; Law et al., 2018). The PLS analysis of the transcriptome and proteome including the arrow plot was done in R version 4.1.0 (R Core Team, 2019) using the mixOmics package (Rohart et al., 2017).

## Results

### Global Changes in the *Msm* Transcriptome and Proteome Upon Zn^2+^ Limitation

**FIGURE 1:**
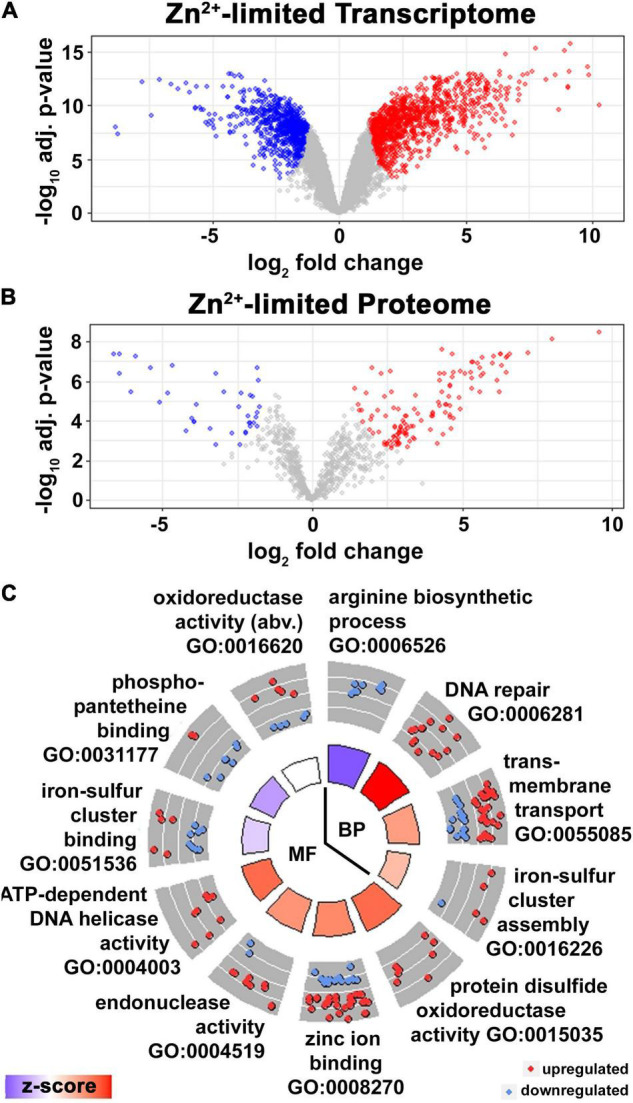
Adaptive response to Zn^2+^ limitation in *Msm*. **(A)** Volcano plot of DE transcripts (absolute lfc > 1, adj. *p*-Value < 0.05) in wild type *Msm* in ZLM vs. ZRM. Transcripts (data points) colored in red, blue, and gray are significantly upregulated, downregulated or not significantly regulated in ZLM vs. ZRM, respectively. Three independent biological replicates for each condition were used in the analysis. **(B)** Volcano plot of DE proteins (absolute lfc > 1, adj. *p*-Value < 0.05) in wild type *Msm* in ZLM vs. ZRM. Proteins (data points) colored in red, blue, and gray are significantly upregulated, downregulated or not significantly regulated in ZLM vs. ZRM, respectively. Three independent biological replicates for each condition were used in the analysis. **(C)** Circle plot showing GO terms of biological processes (BP) and molecular functions (MF) significantly enriched in the DE transcripts in wild type ZLM vs. ZRM (*p*-Value < 0.05). Each slice of the circle is labeled with the enriched GO term, the outer circle shows a scatter plot of lfc values for DE genes with the given GO annotation, where red circles display upregulated and blue circles downregulated genes in wild type ZLM vs. ZRM. The color of bars in the inner circle correlates with the z-score which indicates whether the given biological process is more likely to be increased (red) or decreased (blue) in the dataset and the height of the bars represents the significance for the enriched term (larger bars have smaller *p*-Value). When necessary, the full-length description of some GO terms are abbreviated (abv.) for clarity.

Our first goal was to define the transcriptional response to Zn^2+^ limitation by comparing expression patterns of *Msm* grown in ZLM (i.e., Sauton’s medium without Zn^2+^ supplementation) and ZRM (i.e., Sauton’s medium with standard 6 μM Zn^2+^ supplementation) using high-throughput RNA sequencing (RNA-seq). After removing genes with no (*n* = 139) and low (*n* = 172) expression values across both conditions, we detected 95% of coding and non-coding transcripts in the *Msm* genome. Transcript abundances were analyzed to determine differential expression (DE) patterns using an absolute fold-change cut-off of two (log_2_ fold-change, lfc = 1), yielding 1,084 genes that are upregulated and 742 genes that are downregulated at the transcript level in the Zn^2+^-limited condition (i.e., wild type grown in ZLM vs. wild type grown in ZRM) (Figure 1A). As expected, we observed upregulation of genes in the *Msm* Zur regulon (Novichkov et al., 2013; Supplementary Table S1) and, as observed in *Mtb* (Dow et al., 2021), hundreds of genes not under control of Zur were also upregulated (or downregulated) in the Zn^2+^-limited condition (Supplementary Table S2). Transcriptional changes during gradual Zn^2+^ limiting conditions when bacteria are grown in ZLM (i.e., without addition of a Zn^2+^ chelator) had a strong overlap with previous reports for acute Zn^2+^ depletion through addition of the Zn^2+^-specific chelator TPEN; 74% of DE genes in TPEN-treated *Msm* followed the same expression levels in the wild type ZLM vs. wild type ZRM comparison (Goethe et al., 2020).

In *Mtb*, analysis of the proteome supported the transcriptomic changes observed, and a conserved response to Zn^2+^-limiting conditions was described from the overlap of DE transcripts and proteins (Dow et al., 2021). Taking the same approach here, we probed the proteomes of wild type *Msm* grown in ZRM and ZLM using semi-quantitative spectral counting (SpC) from shotgun proteomics. After removing lowly expressed proteins having fewer than 10 SpC, a total of 804 proteins were identified (Supplementary Table S3). After imparting an absolute cut-off value of two (lfc = 1) to define DE, 162 proteins were found to be DE in the Zn^2+^-limited condition (i.e., wild type grown in ZLM vs. ZRM) (Figure 1B and Supplementary Table S3). Just under 50% of these genes (*n* = 77) had overlap between the expression levels of the protein and the transcript (Supplementary Table S4). To describe the functionality of the DE genes with a conserved response at the level of the transcriptome and proteome in the Zn^2+^-limited condition, we conducted an enrichment analysis for gene ontology (GO) terms. Enriched GO terms yielded processes involved in translation, metal ion transport, oxidoreductase activity and structural constituent of the ribosome. The list of genes with conserved expression levels of the transcript and protein was too short to provide a high-power analysis, so we expanded the enrichment analysis to include all DE proteins. This slightly expanded the analysis with significantly enriched GO terms including processes involved in rRNA binding and N-acetyltransferase activity.

While the overlap of DE transcripts and proteins demonstrates the robust cellular response to Zn^2+^-limiting conditions, the low resolution at the level of the proteome using the shotgun proteomics approach allowed us to evaluate only 9% of the changes detected at the level of the transcriptome. Therefore, to expand the enrichment analysis for GO terms and to provide a greater understanding of the functionality of DE genes in the Zn^2+^-limited condition, we conducted the enrichment analysis on the list of DE transcripts. The enrichment analysis covered 1824 DE genes, resulting in 728 genes in 12 functional annotations (GO terms of “biological processes” and “molecular function”) that were enriched in the Zn^2+^-limited condition (Figure 1C). The circle plot shows downregulation of processes involved in arginine biosynthesis and phosphopantetheine binding during Zn^2+^ limitation. On the other hand, processes involved in DNA maintenance (i.e., DNA repair, endonuclease activity, and DNA helicase activity) as well as protein disulfide oxidoreductase activity were the most upregulated processes during Zn^2+^ limitation. Similar results were apparent in enriched KEGG terms; “homologous recombination” was the most upregulated term, while “alanine, aspartate and glutamate metabolism” and “oxidative phosphorylation” were downregulated terms in wild type ZLM vs. wild type ZRM comparison (Supplementary Figure S1). In conclusion, Zn^2+^ limitation in *Msm* triggers a robust transcriptional response, including genes that are required for redox homeostasis and repairing damaged proteins and DNA. The transcriptomic response is reflected in the proteome, however, less than half of the DE proteins positively correlated with the transcriptome suggesting post-transcriptional or translational processes may significantly contribute to the cellular response to Zn^2+^-limitation.

### Upregulated Oxidative Stress Response Is Conserved in Zn^2+^-Limited Mycobacteria

**FIGURE 2:**
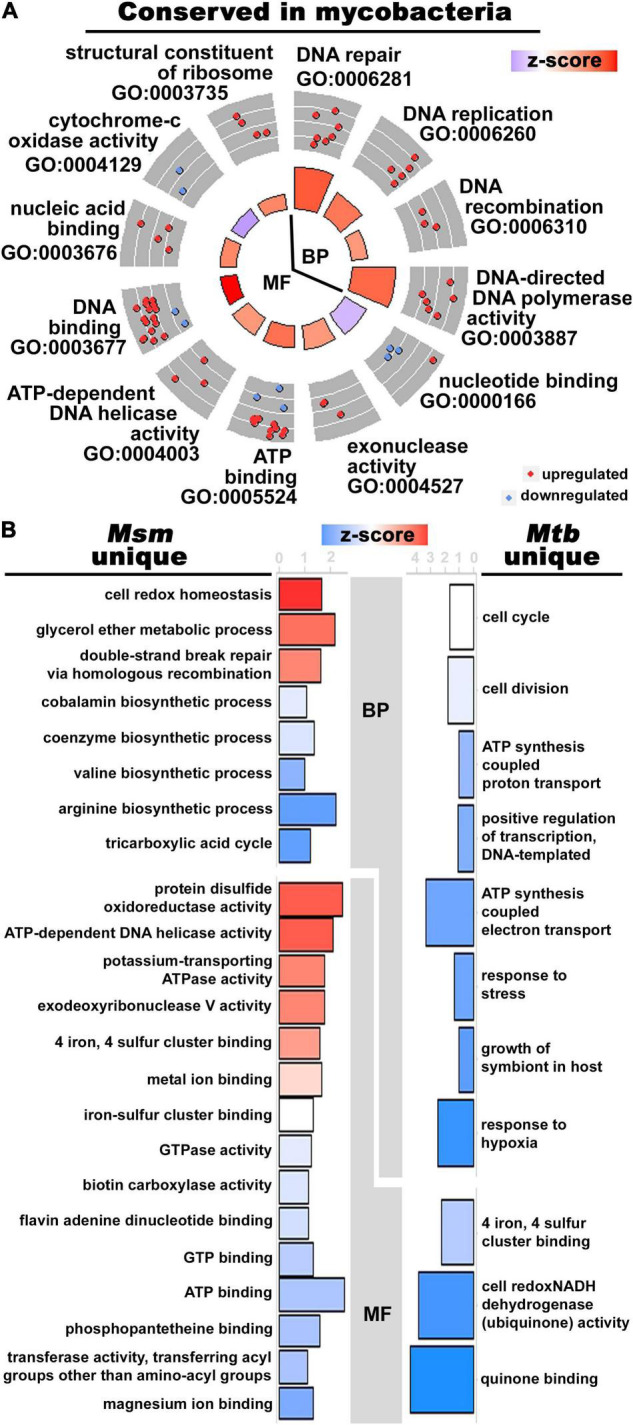
The alignment and discordance of DE orthologous genes in Zn^2+^-limited *Msm* and *Mtb*. **(A)** Circle plot showing enriched GO terms in the orthologous genes with shared differential expression patterns in response to Zn^2+^ limitation in mycobacteria. The enrichment was conducted using the locus tags from *Msm*. See Figure 1C for circle plot explanation. **(B)** Inflected vertical bar graphs of enriched GO terms in the orthologous mycobacterial genes that were uniquely regulated in Zn^2+^-limited *Msm* (left) and *Mtb* (right). The enrichments were conducted using the locus tags of the respective mycobacterial species (i.e., MSMEG tags for unique DE genes in *Msm* and Rv tags for unique DE genes in *Mtb*). The bars are colored by z-score as explained for the circle plot in Figure 1C. The axis of each bar graph is the -log_10_ (*p*-Value) of the enriched term. Due to the restriction of only considering orthologous genes, significantly enriched GO terms were defined with *p*-Value < 0.1 for all analyses in this figure.

The concentration of Zn^2+^ available during growth has a drastic impact on the transcriptomes of mycobacteria; *Msm* differentially regulated 26% and *Mtb* regulated 17% of their respective transcriptomes during Zn^2+^-limiting conditions. The transcriptomics analysis of Zn^2+^-limited *Msm* yielded similar results to those described for Zn^2+^-limited *Mtb*, specifically regarding increased DNA repair processes (Dow et al., 2021). Over half of the genes in *Mtb* have orthologues in *Msm*; 403 of the orthologous genes were DE in Zn^2+^-limited *Mtb* (Dow et al., 2021). Of those genes, 41% (*n* = 167) were also DE in *Msm*, but only 30% (*n* = 122) were DE in the same direction (i.e., up- or down- regulated) in both species in the Zn^2+^-limited condition (Supplementary Table S5). An enrichment of GO terms in the orthologous DE genes with the same regulation level in *Msm* and *Mtb* highlighted that processes involved in DNA replication, recombination and repair were upregulated during Zn^2+^ limitation in both mycobacteria (Figure 2A and Supplementary Table S5). The response to damaged DNA is further implicated by upregulation of the gene encoding the DNA-damage sensing alternative sigma factor SigG (Smollett et al., 2011) in both *Msm* (MSMEG_0219) and *Mtb* (Dow et al., 2021; Supplementary Table S5). Furthermore, like *Mtb* (Dow et al., 2021), *Msm* also upregulates key antioxidant enzymes including catalase-peroxidase (*katG*, MSMEG_6384), alkylhydroperoxidase (*ahpC*, MSMEG_4891) and thioredoxin-disulfide reductase (*trxB*, MSMEG_6933) in Zn^2+^-limited conditions, the latter two being upregulated at the protein level as well (Supplementary Tables S3, S5). The shift from fatty acid biosynthesis to degradation and decreased oxidative phosphorylation described in *Mtb* (Dow et al., 2021) was also evident in *Msm* when projecting DE genes belonging to the KEGG term “metabolic pathways” onto a global metabolic network (Supplementary Figure S2). In sum, the overlap in the transcriptomics analysis of Zn^2+^-limited *Msm* with a comparable analysis in *Mtb* (Dow et al., 2021) highlights conservation of the upregulated oxidative stress response during Zn^2+^-limiting conditions in mycobacteria.

### Unique Regulation of Certain Processes in Zn^2+^-Limited Mycobacteria

While there was an overlap of specific processes in Zn^2+^-limited *Msm* and *Mtb* (Dow et al., 2021), the expression patterns of genes uniquely DE in either mycobacteria experiencing Zn^2+^-limitation was much greater than those that were shared. To begin, 442 orthologous genes were DE in *Msm*, but were not DE in *Mtb* (Dow et al., 2021; Supplementary Table S6). Enriched GO terms show approximately equal distribution of processes that are up- and down- regulated in this list of genes. Upregulated processes unique to Zn^2+^-limited *Msm* include redox homeostasis, protein disulfide oxidoreductase activity and processes involved with DNA repair (Figure 2B), possibly indicating a more robust oxidative stress response in Zn^2+^-limited *Msm* compared to *Mtb*. Downregulated processes include arginine and valine biosynthesis, the tricarboxylic acid cycle, and genes involved in binding to magnesium, ATP and GTP (Figure 2B).

There were 236 DE orthologous genes in *Mtb* (Dow et al., 2021) that were not DE in *Msm*, and 5 of these genes were not detected (i.e., did not pass low expression filter) in *Msm* (Supplementary Table S7). Among these genes, 64% are downregulated and 36% are upregulated in the Zn^2+^ limited condition in *Mtb* (Dow et al., 2021), however, the enriched processes associated with these genes are exclusively downregulated (Figure 2B and Supplementary Table S7). The functionally enriched genes are involved in processes of quinone binding, NADH dehydrogenase activity, growth of symbiont in host, and the response to hypoxia and stress (Figure 2B). Notably, the two-component response regulator *mtrA* and four genes in the mammalian cell entry operon 1 (*mce1C-F*) were uniquely downregulated in Zn^2+^-limited *Mtb* (Supplementary Table S7). A large portion of the uniquely regulated genes in *Mtb* are directly involved in processes relevant to the host-pathogen interaction.

In addition to the uniquely DE genes described above, 45 orthologous genes were discordantly regulated in Zn^2+^-limited *Msm* and *Mtb* (Dow et al., 2021; Supplementary Table S8). Enriched GO terms in the genes that were downregulated in *Msm* but upregulated in *Mtb* (Dow et al., 2021) were involved in “*de novo*” UMP and pyrimidine biosynthesis, and arginine biosynthesis (Supplementary Figure S3). On the other hand, enriched GO terms that were upregulated in *Msm* but downregulated in *Mtb* (Dow et al., 2021) include processes involved in ergothioneine biosynthesis from histidine, oxidoreductase activity and ribonucleoside-diphosphate reductase activity using thioredoxin disulfide as acceptor (Supplementary Figure S3). Genes with the most remarkable levels of discordant regulation include ferredoxin, i.e., *fdxA* (MSMEG_1124) and lipase, i.e., *lipU* (MSMEG_5271), which were both significantly downregulated in *Mtb* (Dow et al., 2021), but upregulated in *Msm* (Supplementary Table S8). Furthermore, the gene encoding the WhiB6 transcription factor is highly upregulated in *Mtb* (Dow et al., 2021), but not DE in *Msm* (MSMEG_0051), whereas the opposite is true for WhiB7 (MSMEG_1953) (Supplementary Tables S6, S7). Altogether, these results suggest that, in response to Zn^2+^-limitation, mycobacteria may have evolved unique regulation strategies where *Mtb* regulates processes relevant to the context of infection and *Msm* regulates processes to support cell growth such as maintaining redox homeostasis, repairing damaged biomolecules and modulating central carbon metabolism.

### *Msm* Regulates Central Carbon Metabolism During Zn^2+^ Limitation

It was curious to see differential regulation of many genes involved in central metabolism in Zn^2+^-limited *Msm*. A general downregulation of metabolic genes might be anticipated in Zn^2+^-limited *Msm* since cell density is much higher in ZRM than ZLM (Dow and Prisic, 2018), a phenotype not observed in *Mtb* (Dow et al., 2021), but this was not the trend observed. In fact, of the DE genes belonging to metabolic pathways as defined by KEGG, 40% are upregulated and 60% are downregulated in the Zn^2+^-limited condition. Analysis of the DE genes involved in carbon metabolism showed a specific downregulation of genes encoding enzymes involved in the non-oxidative arm of the pentose phosphate pathway (PPP) along with two downstream enzymes involved in glycolysis, glyceraldehyde-3-phosphate dehydrogenase (GAPDH), i.e., *gap* (MSMEG_3084) and phosphoglycerate kinase, i.e., *pgk* (MSMEG_3085), during Zn^2+^ limitation (Supplementary Figure S4). The oxidative arm of the PPP (ox-PPP) is the predominant producer of cellular NADPH; redirection of glycolytic flux through the ox-PPP can increase the antioxidant defenses that combat oxidative stress (Mullarky and Cantley, 2015). Redirection of glycolytic flux through the ox-PPP can also be achieved by inhibition of downstream glycolytic enzymes such as GAPDH (Mullarky and Cantley, 2015). The changes in expression patterns of genes that divert glycolytic flux through the ox-PPP for increased NADPH production in Zn^2+^-limited *Msm* further implicate the relevance of combating oxidative stress in the Zn^2+^-limited environment.

### Deletion of the *altRP* Operon Significantly Impacts the Transcriptome and Proteome

**FIGURE 3:**
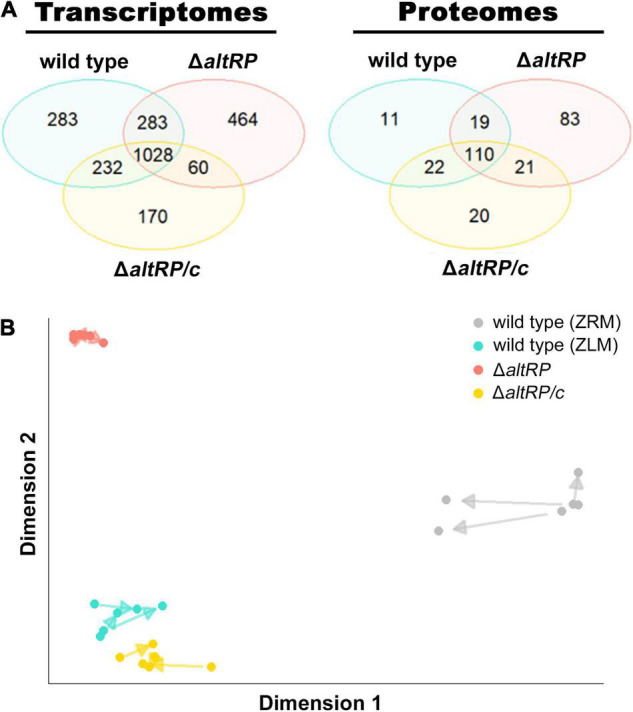
Transcriptomes and proteomes of Zn^2+^-limited wild type, Δ*altRP* and Δ*altRP/c* compared to the Zn^2+^-replete wild type. **(A)** Venn diagrams showing the overlap of DE genes (left) and proteins (right) in the wild type, Δ*altRP*, and Δ*altRP*/c (grown in ZLM) when compared to the wild type grown in ZRM. **(B)** Arrow plot from PLS analysis of the transcriptomes and proteomes of the wild type, Δ*altRP*, Δ*altRP*/c (grown in ZLM) and the wild type grown in ZRM. The representation space maximizes the covariance of the components, and as such the scale is arbitrary and absolute values do not affect the alignment.

*Msm* grown in ZLM build active Alt ribosomes (i.e., ribosomes containing AltRPs), and AltRPs are required for the morphogenesis of Zn^2+^-limited *Msm* (Dow and Prisic, 2018; Tobiasson et al., 2019). Since the Δ*altRP* mutant grows in ZLM, but is unable to grow in the presence of TPEN (Dow and Prisic, 2018), it is possible to investigate the involvement of AltRPs during the response to gradual Zn^2+^ limitation in ZLM. To specify the role of AltRPs in the Zn^2+^-dependent phenotype, we investigated the transcriptomes of the Δ*altRP* mutant and the *in trans* complement strain (Δ*altRP/c*) grown in ZLM compared to the wild type grown in ZRM (Supplementary Table S9). Principal component analysis (PCA) shows that Zn^2+^ is the leading dimension explaining 71% of variance in the transcriptomics dataset, while genotype (i.e., deletion of AltRPs) explains only 18% (Supplementary Figure S5). As in the wild type ZLM vs. wild type ZRM comparison, we defined DE using a lfc = 1 cut-off, resulting in 1,835 and 1,490 DE genes in Δ*altRP* and Δ*altRP/c* when compared to the wild type grown in ZRM, respectively (Supplementary Table S9). To highlight gene expression patterns that are affected by deletion of AltRPs (i.e., the *altRP* operon), we looked for overlap in DE genes of the wild type, Δ*altRP* and Δ*altRP/c* strains grown in ZLM, compared to the wild type grown in ZRM. The Venn diagram in Figure 3A shows that 56% of the DE genes in the wild type ZLM vs. wild type ZRM comparison were also DE in the Δ*altRP* and Δ*altRP/c* strains (*n* = 1028). This data shows, in agreement with the PCA, that majority of DE genes are driven by Zn^2+^ concentration and not the presence or absence of AltRPs.

Because of the potential role for AltRPs in translation (Chen et al., 2020), the next step was to define the correlation between the transcriptome and proteome of Zn^2+^-limited *Msm*, and to determine if the Δ*altRP* mutant follows patterns observed in the wild type. Accordingly, in addition to probing the proteomes of the wild type grown in ZRM and ZLM, we also included the Δ*altRP* mutant and its complement strain (Δ*altRP/c*) grown in ZLM. After removing proteins with fewer than 10 SpC across all strains and imparting an absolute cut-off value of two (lfc = 1) to define DE, we identified 233 and 173 DE proteins in Δ*altRP* and Δ*altRP/c* respectively, when compared to the wild type grown in ZRM (Supplementary Table S3). Again, Zn^2+^ is the leading dimension describing 63% and genotype accounting for 19% of variance in the dataset (Supplementary Figure S5). The Venn diagram in Figure 3A shows the overlap of DE proteins in the Zn^2+^-limited strains compared to the wild type grown in ZRM. Similar to the transcriptomes, most DE proteins (68%, *n* = 110) in the wild type ZLM vs. wild type ZRM comparison were conserved across all Zn^2+^-limited strains (i.e., wild type, Δ*altRP* and Δ*altRP/c*), but again, the Δ*altRP* mutant had a considerable number of DE proteins (*n* = 83) unique to this comparison.

A multivariate analysis using partial least squares (PLS) regression, which maximizes the covariance between components from two datasets (e.g., transcriptomics and proteomics) (Wold et al., 2001), was used to create an arrow plot overlapping the covariance together in one dimension. In the arrow plot, each arrow connects the transcriptome (tail) and the proteome (head) from the same sample. Shorter arrows indicate better agreement found by PLS between both datasets. From the arrow plot in Figure 3B, we see that for all strains, the proteome is closely related to the transcriptome and there is preservation of the dimensions driving variance in both datasets. Notably, while the Zn^2+^-replete condition had the most variation between the transcriptome and proteome, the Δ*altRP* mutant had the least amount of variation among the Zn^2+^-limited samples, which may indicate altered processes of post-transcriptional or post-translational regulation in the Δ*altRP* mutant.

### Discordance Between Transcript and Protein Abundance in the Wild Type and the Δ*altRP* Mutant

Based on the multi-omics analysis and given the potential role for AltRPs in the process of translation (Chen et al., 2020), we wanted to further investigate the variance between the transcriptome and proteome with respect to AltRP expression. To this end, we investigated examples of discordant regulation between a transcript and the protein it encodes in the wild type ZLM vs. wild type ZRM comparison (i.e., in wild type cells with Alt ribosomes). In our dataset, discordant regulation could be observed in one of two ways, either the protein is underrepresented compared to its transcript, or the protein is overrepresented compared to its transcript. Genes with underrepresented proteins could have discordant regulation levels in one of the following three ways: [1] the transcript is upregulated and the protein is not DE (*n* = 68), [2] the transcript is upregulated and the protein is downregulated (no examples) or [3] the transcript is not DE and the protein is downregulated (*n* = 24) (Supplementary Table S10). On the other hand, genes where the protein is overrepresented compared to its transcript could have discordant regulation levels in one of the following three ways: [1] the transcript is downregulated and the protein is not DE (*n* = 82), [2] the transcript is downregulated and the protein is upregulated (*n* = 5) or [3] the transcript is not DE and the protein is upregulated (*n* = 55) (Supplementary Table S11). In all these comparisons, the gene was only considered if both the transcript and the protein were detected.

Next, to divulge to role of AltRPs in discordant gene expression, we turned to look at expression patterns of the genes described above in the Δ*altRP* vs. wild type grown in ZRM comparison. If an Alt ribosome-dependent regulation (e.g., selective translation by Alt ribosomes) is responsible for a discordant regulation of gene products, we would expect that the Δ*altRP* strain would not have this discordance. Starting with the proteins that are underrepresented compared to their transcripts, we found that more than a half of them follow the same discordance in the wild type and the Δ*altRP*, and therefore are not candidates for Alt-dependent translational regulation (Supplementary Table S10). Moreover, one-fifth of the genes were not transcriptionally regulated in the same way in the wild type and the Δ*altRP* mutant, and most of these genes were not DE in the Δ*altRP* mutant, so comparison of transcript-protein abundances for these genes was inconclusive. However, a quarter of the genes had discordant regulation in the wild type that could be attributed to the presence of AltRPs. One set of genes had significantly upregulated transcripts in both the wild type and Δ*altRP* mutant, and although at the protein level they were not DE in the wild type, they were significantly upregulated in the Δ*altRP* mutant (Table 1). Notable proteins on this list are involved in Fe-S assembly (SufC/D), Clp protease (ClpP) and catalase peroxidase (KatG). Another set of genes included nine examples that were not DE at the transcript level in wild type or Δ*altRP*, and were significantly downregulated at the protein level in wild type, but were not DE in Δ*altRP* (Table 1). Interestingly, this set of genes encodes five ribosomal proteins and two proteins involved in translation (IF-2 and DnaJ chaperone).

**TABLE 1:** Genes with discordant regulation between the transcript (RNA) and protein (Prot.) in Zn^2+^-limited wild type (ZLM) and the overlap with Δ*altRP*.

				*wild type* (ZLM)		Δ*altRP* (ZLM)	
				
Type of discordance	Locus tag	Gene	Description	lfc RNA	lfc Prot.	lfc RNA	lfc Prot.
Under-represented protein	MSMEG_0117		hydrolase	5.62	not DE	7.89	4.45
	MSMEG_0118		conserved hypothetical protein	4.25	not DE	5.29	4.90
	MSMEG_0121		rhamnolipids biosynthesis 3-oxoacyl-[acyl-carrier-protein] reductase	2.21	not DE	2.44	1.88
	MSMEG_0505		probable sugar ABC transporter, substrate-binding protein, putative	2.87	not DE	3.87	5.84
	MSMEG_1419		conserved hypothetical protein	6.20	not DE	4.60	2.30
	MSMEG_1652		O-acetylhomoserine sulfhydrylase	1.66	not DE	1.65	1.84
	MSMEG_2839		transcriptional accessory protein	2.04	not DE	2.48	3.09
	MSMEG_3123	*sufD*	FeS assembly protein SufD	1.60	not DE	2.59	4.77
	MSMEG_3124	*sufC*	FeS assembly ATPase SufC	1.24	not DE	2.75	2.30
	MSMEG_3127		conserved protein, DUF59	1.42	not DE	2.36	3.13
	MSMEG_4673	*clpP*	Clp protease	2.12	not DE	2.90	2.19
	MSMEG_5102		ABC transporter ATP-binding protein	3.96	not DE	6.09	5.22
	MSMEG_5119	*pruA*	1-pyrroline-5-carboxylate dehydrogenase	2.02	not DE	1.67	4.63
	MSMEG_6384	*katG*	catalase/peroxidase HPI	4.65	not DE	5.70	4.28
	MSMEG_0069	*infB*	translation initiation factor IF−2 protein	not DE	−2.69	not DE	not DE
	MSMEG_1443	*rplP*	ribosomal protein L16	not DE	−3.20	not DE	not DE
	MSMEG_1468	*rpsN*	ribosomal protein S14p/S29e	not DE	−3.92	not DE	not DE
	MSMEG_1525		50S ribosomal protein L17	not DE	−1.86	not DE	not DE
	MSMEG_3244		hypothetical protein	not DE	−2.21	not DE	not DE
	MSMEG_4282		conserved hypothetical protein	not DE	−1.82	not DE	not DE
	MSMEG_4504	*dnaJ*	chaperone protein DnaJ	not DE	−2.05	not DE	not DE
	MSMEG_6209		conserved hypothetical protein	not DE	−2.06	not DE	not DE
	MSMEG_6895	*rpsR*	ribosomal protein S18	not DE	−2.94	not DE	not DE
Over-represented protein	MSMEG_0640		oligopeptide transport ATP-binding protein OppD	−1.64	4.36	−1.31	not DE
	MSMEG_3044		dihydroorotase	−2.07	not DE	−2.28	3.75
	MSMEG_3599		sugar-binding transcriptional regulator, LacI family protein	−1.65	not DE	−1.31	1.69
	MSMEG_3630		transcriptional repressor, CopY family protein	−2.05	not DE	−2.65	2.14
	MSMEG_1812		conserved hypothetical protein	−1.74	not DE	−1.58	−4.11
	MSMEG_2261		hypothetical protein	−3.14	not DE	−4.85	−5.42
	MSMEG_3771	*argR*	arginine repressor	−2.35	not DE	−2.29	−4.66
	MSMEG_2389	*hup*	DNA-binding protein HU	−3.75	not DE	−2.25	−1.57
	MSMEG_5520		conserved hypothetical protein	−1.98	not DE	−1.78	−2.32
	MSMEG_3443		hypothetical protein	−1.93	not DE	−2.02	−1.86
	MSMEG_1548		propanediol utilization: dehydratase, medium subunit	−1.71	not DE	−4.63	−2.47
	MSMEG_2616	*cobO*	cob(I)alamin adenosyltransferase	−1.63	not DE	−1.87	−3.17
	MSMEG_4261		ubiquinol-cytochrome c reductase cytochrome c subunit	−2.19	not DE	−2.96	−5.17
	MSMEG_0114		extracellular solute-binding protein, family protein 3	not DE	1.99	not DE	not DE
	MSMEG_2081		putative acyl-CoA dehydrogenase	not DE	2.65	not DE	not DE
	MSMEG_3065	*sun*	ribosomal RNA small subunit methyltransferase B	not DE	2.42	not DE	not DE
	MSMEG_3302		short-chain dehydrogenase/reductase SDR	not DE	4.43	not DE	not DE
	MSMEG_4278	*gcvT*	glycine cleavage system T protein	not DE	2.04	not DE	not DE
	MSMEG_6096		Bvg accessory factor family protein	not DE	3.17	not DE	not DE
	MSMEG_6101	*folK*	2-amino-4-hydroxy-6-hydroxymethyldihydropteridine pyrophosphokinase	not DE	2.50	not DE	not DE
	MSMEG_6309		ABC transporter, ATP-binding protein	not DE	3.37	not DE	not DE
	MSMEG_6422		ferritin family protein	not DE	2.42	not DE	not DE

*Only genes for which the ΔaltRP mutant followed the same regulation as the wild type (ZLM) at the transcript level are shown. The lfc values for DE transcripts and proteins are given and “not DE” indicates that the transcript or protein was not significantly differentially expressed. Both comparisons in this table (i.e., wild type and ΔaltRP) are compared to the Zn^2+^-replete wild type (ZRM).*

Moving on to the genes where the protein was overrepresented compared to its transcript in the wild type ZLM vs. wild type ZRM comparison, we also found that half of these genes follow the same discordant regulation levels in Δ*altRP* vs. wild type ZRM (Supplementary Table S11). Another one-third of the genes did not follow the same regulation at the transcript level in the Δ*altRP* mutant as observed in the wild type grown in ZLM, again making the comparison of AltRP-dependent discordant expression for these genes inconclusive (Supplementary Table S11). The final one-sixth of the genes had discordant regulation that could be attributed to AltRPs (Table 1). With a few exceptions, the regulation levels in the Δ*altRP* mutant mirrored the wild type but did not meet the threshold for significance (Supplementary Table S11). Notable examples include the ABC transporter ATP-binding protein (MSMEG_6309), which was significantly overrepresented at the protein level in the wild type but not in the Δ*altRP* mutant, and the gene encoding the oligopeptide transport ATP-binding protein OppD (MSMEG_0640), showing strong upregulation at the protein level only in the wild type. Interestingly, these proteins that are significantly overrepresented only in the wild type background are involved in transporting molecules into the cell.

Of note in these comparisons, is that about half of the discordant examples of regulation levels were preserved in the Δ*altRP* background, and there were relatively few examples where the discordance found in wild type was drastically different in the Δ*altRP* background. Additionally, many of these genes did not agree at the transcript level between wild type and Δ*altRP*. In nearly all these cases, the protein expression level followed that of the transcript in the Δ*altRP* mutant. These results, along with those presented in the arrow plot in Figure 3B, indicate that changes in protein expression patterns between the wild type and the Δ*altRP* mutant are strongly driven by alterations in the transcriptomes of these two strains. While we did identify potential examples of regulation by Alt ribosomes through investigation of discordant expression levels (Table 1), they were relatively rare and are probably not directly and/or solely responsible for the different phenotypes observed in the wild type and the Δ*altRP* mutant experiencing Zn^2+^ limitation.

### Increased Oxidative Stress Response During Zn^2+^ Limitation Is Independent of AltRP Expression

**FIGURE 4:**
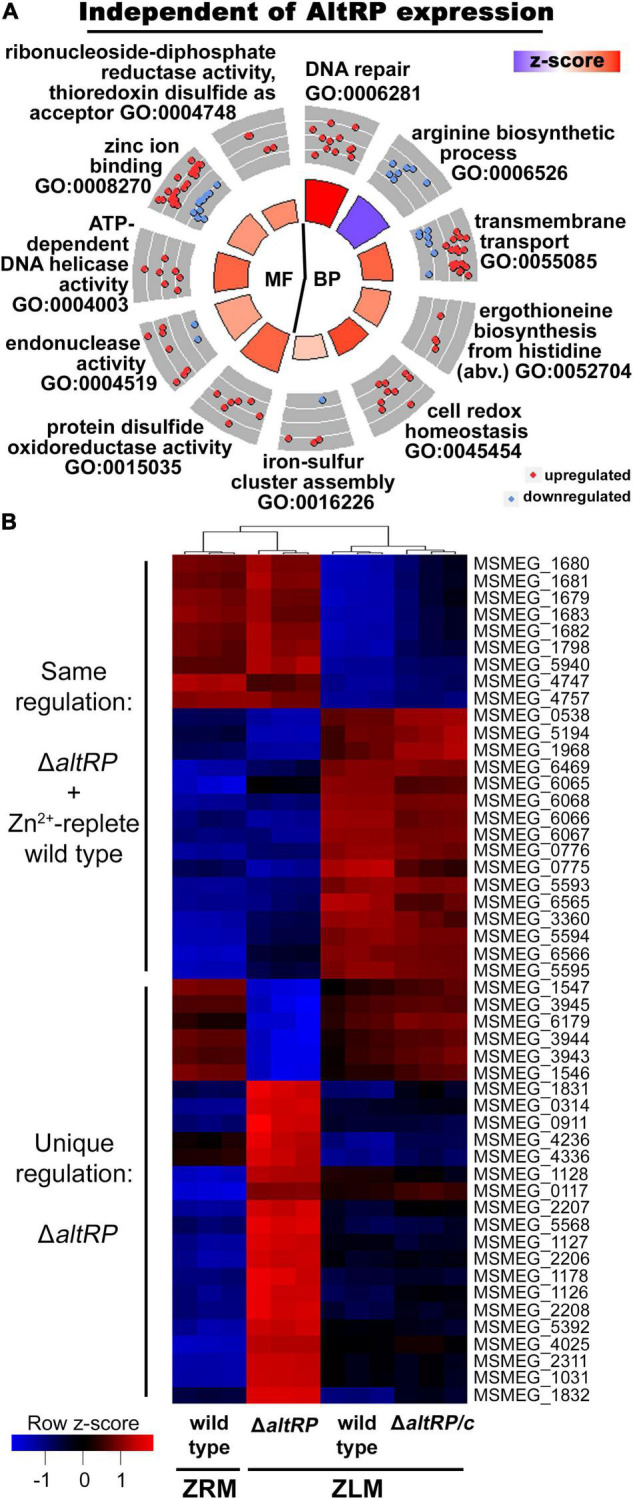
Differentially expressed genes independent **(A)** and dependent **(B)** of AltRP expression during Zn^2+^-limitation. **(A)** Circle plot showing enriched GO terms in the DE genes shared between the wild type and the Δ*altRP* mutant during Zn^2+^ limitation (i.e., both grown in ZLM and compared to the wild type grown in ZRM). DE analysis was conducted on three independent biological replicates for each strain. See Figure 1C for circle plot explanation. **(B)** Heatmap showing relative transcript expression levels in all strains and conditions for the top 50 DE genes (by adj.P.val) between the Δ*altRP* vs. wild type in ZLM comparison. For each strain or condition, the relative expression for each independent biological replicate is shown in the column.

From the multi-omics analysis of the wild type and the Δ*altRP* mutant, we observed that deletion of AltRPs causes a significant perturbation to the transcriptome, which partially, correlates with changes to the proteome. However, the Venn diagrams in Figure 3A also show a conserved response to Zn^2+^ limitation in the wild type, Δ*altRP* mutant and the complement strain (Δ*altRP/c*). Like the observed overlap in transcript and protein expression levels in the wild type, just over 50% of the proteins with concordant regulation in all strains (i.e., at the intersection of all strains in both Venn diagrams in Figure 3A) overlapped with concordantly regulated transcripts in all strains (Supplementary Table S12). Again, pathway analysis using proteomics was not possible due to limited resolution, so we used transcriptomics data to understand which processes employed during Zn^2+^ limitation are independent of AltRP expression. We selected the DE genes common to all three strains grown in ZLM when compared to the wild type grown in ZRM (union of all comparisons) from the Venn diagram in Figure 3A (*n* = 1028) to conduct an enrichment analysis for GO terms. The enrichment analysis covered 1027 shared DE genes, yielding 421 genes with 11 functional annotations of “biological processes” and “molecular functions” that were significantly enriched in all strains grown in ZLM (regardless of deletion of the *altRP* operon) when compared to the wild-type Zn^2+^-replete condition (Figure 4A).

As predicted by the fact that over half of the DE genes in the wild type ZLM vs. wild type ZRM comparison were preserved across all Zn^2+^-limited strains, the GO enrichment analysis has significant overlap with DE genes in Zn^2+^-limited wild type (Figures 1C, 4A). In all Zn^2+^-limited strains, independent of AltRP expression, processes involved in DNA repair and iron-sulfur cluster assembly are upregulated, and the arginine biosynthetic process is downregulated (Figure 4A). Additionally, key antioxidant enzymes upregulated in Zn^2+^-limited wild type were also upregulated in Δ*altRP* and Δ*altRP/c*, including catalase-peroxidase (*katG*), alkylhydroperoxidase (*ahpC*) and thioredoxin (*trx* and *trxB*) (Supplementary Table S9). Among these genes, *ahpC*, *trx*, and the DNA repair protein *recA* were DE at the level of the transcriptome and proteome in all Zn^2+^-limited strains (Supplementary Table S3). The GO enrichment of DE genes shared between the wild type, Δ*altRP* and Δ*altRP/c* strains in Zn^2+^-limiting conditions when compared to the Zn^2+^-replete wild type (i.e., wild type grown in ZRM), and the overlap of specific genes at the protein level, shows that the increased oxidative stress response upon Zn^2+^-limiting conditions occurs independently of AltRP expression in *Msm*.

### AltRP-Dependent Response During Zn^2+^-Limitation Includes Downregulated Cell Division and Nucleotide Biosynthesis and Altered Respiration

Beyond the conserved response to Zn^2+^ limitation in all strains, the multi-omics analysis shows that the *altRP* operon deletion did impart significant changes at the level of the transcriptome and proteome. To further define the impact of lacking AltRPs during Zn^2+^ limitation, we investigated the DE genes that were not conserved between the Δ*altRP* mutant and the wild type in ZLM. First, from the Venn diagram of transcripts in Figure 3A, we see the Δ*altRP* mutant has many unique DE genes, but also, nearly as many genes are only DE in the wild type and Δ*altRP/c* strains. The 232 genes at the intersection of the wild type and Δ*altRP/c* comparisons in Figure 3A were interesting because they represented processes that were only DE in the Zn^2+^-limited condition when AltRPs are present (Supplementary Table S13). Genes that are exclusively downregulated in Alt ribosome-containing cells are involved in processes of [1] cell division including the chromosomal replication initiator protein *dnaA* (MSMEG_6947), *sepF* (MSMEG_4219), *pbpB* (MSMEG_4233) and *amiB* (MSMEG_1679), [2] respiration through succinate dehydrogenase, *sdhCD* (MSMEG_1671 and MSMEG_1672), and [3] purine nucleotide biosynthesis, *purA* (MSMEG_0759), *nadBC* (MSMEG_3200 and MSMEG_3201) and *aspA* (MSMEG_1677). Genes exclusively upregulated in Alt ribosome-containing cells are involved in [1] respiration, through NADH dehydrogenase complex, *nuoB* and *nuoE* subunits (MSMEG_2062 and MSMEG_2059) and the non-proton pumping (type II) NADH dehydrogenase protein *ndh* (MSMEG_3621), and [2] regulation of gene expression including the SigE anti-sigma factor *rseA* (MSMEG_5071) and the SigH anti-sigma factor *rshA* (MSMEG_1915). All in all, processes associated with cell division and nucleotide biosynthesis are downregulated and respiration is altered only in the presence of AltRPs during Zn^2+^ limitation.

### AltRP-Expressing Cells Alter Nucleotide Acquisition Strategy Through Induction of GOGAT and Decreased Expression of Purine Import Mechanisms

The Venn diagrams in Figure 3A demonstrate unique regulation of a set of genes and proteins in the wild type and the complement, but not the Δ*altRP* mutant in Zn^2+^-limiting conditions. Additionally, among the Zn^2+^-limited strains, the Δ*altRP* mutant also has many unique DE genes, compared to the other two strains that express AltRPs. This led us to investigate the direct effects of lacking AltRP expression in a Zn^2+^-limited environment by comparing transcriptomes of the Δ*altRP* mutant vs. wild type in ZLM which yielded 472 DE genes, with 61% being complemented in Δ*altRP*/c (Supplementary Table S14). The heatmap in Figure 4B shows the relative transcript expression levels in all strains and conditions for the top 50 DE genes between the Δ*altRP* vs. wild type in ZLM, all of these genes were complemented in Δ*altRP/c*. Two patterns emerge in the heatmap (Figure 4B); a set of genes that are either up- or down- regulated uniquely in the Δ*altRP* compared to all other strains (including wild type grown in ZRM) (*n* = 25), and a set of genes that are coordinately up- or down- regulated in the Δ*altRP* mutant and the Zn^2+^-replete wild type (grown in ZRM) when compared to the wild type and Δ*altRP/c* grown in ZLM (*n* = 25) (Supplementary Table S15).

Since the Δ*altRP* phenotype more closely resembled that of the Zn^2+^-replete wild type (Dow and Prisic, 2018), we first looked at the DE genes from the heatmap that are shared between the Δ*altRP* mutant and wild type grown in ZRM (Figure 4B and Supplementary Table S15). In this list, five of the DE genes correlated with DE proteins (all have concordant regulation levels), including the altRP operon and a MarR regulatory protein (MSMEG_0538). Many of the topmost differentially regulated genes are ordered, and thus appear to be in operons (64%), and three genes are transcriptional regulators (*n* = 3). A set of ordered genes that were downregulated in Δ*altRP* and the wild type grown in ZRM encode pyruvate dehydrogenase (MSMEG_5593), ferredoxin-dependent glutamate synthase, *aka* GOGAT (MSMEG_5594) and the MarR transcriptional regulatory protein (MSMEG_5595). A set of ordered genes that were upregulated in Δ*altRP* and the wild type grown in ZRM encodes an amidase (MSMEG_1679), a cytosine/purine/uracil/thiamine/allantoin permease family protein (MSMEG_1683) and an endoribonuclease L-PSP superfamily protein (MSMEG_1681). The L-PSP superfamily belongs to the widely distributed YER057c/YjgF/UK114 family of proteins conserved among all domains of life and serving myriad functions; with a proposed role in purine regulation in bacteria (Sinha et al., 1999). Interestingly, these processes converge at the level of nucleotide metabolism; GOGAT shifts the fate of glutamine nitrogen away from the anaplerotic reactions of the TCA cycle to nucleotide biosynthesis, while import mechanisms for nucleotide precursors are differentially regulated. Together, the expression patterns unique to strains that build primary ribosomes (i.e., Δ*altRP* and the wild type grown in ZRM), suggests that AltRP expression alters the route to nucleotide acquisition.

### Glyoxylate Shunt Is Induced via Expression of Isocitrate Lyase in the Δ*altRP* Mutant

Finally, the Δ*altRP* mutant displayed a set of genes in the heatmap that were DE only in this genetic background (Figure 4B and Supplementary Table S15). Again, many of the genes appear to be in operons and four of the genes are transcriptional regulators. At the protein level, six DE proteins were detected and five were DE and correlated concordantly with DE genes; one of the DE proteins was not complemented in Δ*altRP/c* (MSMEG_0117) (Supplementary Table S15). The Δ*altRP* mutant increased expression of isocitrate lyase (MSMEG_0911), also DE at the protein level, and glucose-6-phosphate 1-dehydrogenase (MSMEG_0314) (Supplementary Table S15). Downregulated expression of coenzyme B12-dependent glycerol dehydrogenase was observed at the transcript (MSMEG_1546 and MSMEG_1547) and protein level (MSMEG_1546) (Supplementary Table S15). Projecting the DE genes involved in carbon metabolism onto a network shows that, in addition to the selective downregulation of the non-oxidative arm of the PPP observed in the wild type, the Δ*altRP* mutant also upregulates key enzymes of the oxidative arm (Supplementary Figure S6). Additionally, the two-component response regulator DevR (MSMEG_3944) was strongly downregulated in the Δ*altRP* mutant at both the transcript and protein level (Supplementary Table S15). All in all, deletion of AltRPs influenced expression patterns of genes involved in central carbon metabolism, including induction of the glyoxylate shunt through isocitrate lyase, and numerous transcriptional regulators during Zn^2+^ limitation.

## Discussion

**FIGURE 5:**
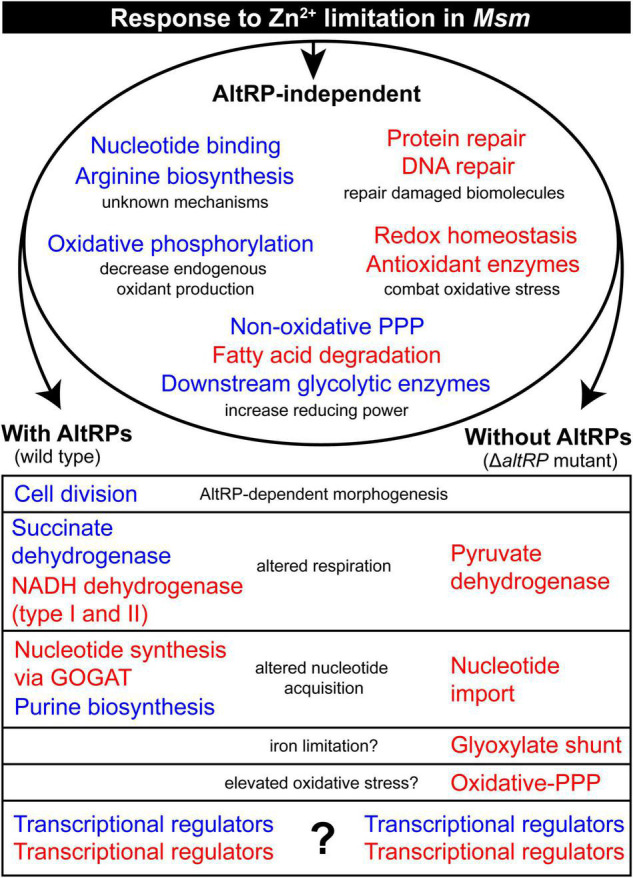
Summary of the molecular response to Zn^2+^-limitation in *Msm*, including AltRP-specific effects. The model shows the shared and unique physiological response of the wild type and the Δ*altRP* mutant grown under Zn^2+^-limiting conditions when compared to the Zn^2+^-replete wild type. The multi-omics data was simplified into general biological processes which are colored in red and blue to indicate up-regulation and down-regulation of process during Zn^2+^ limitation, respectively. The descriptors in black give the presumed reason for the differential regulation of the process in the Zn^2+^-limited environment, or the relevance of the differences between the wild type and Δ*altRP* mutant.

This multi-omics analysis of Zn^2+^-limited *Msm* shows that Zn^2+^ limitation dramatically impacts the transcriptome and proteome while highlighting the contribution of alternative ribosomal proteins (AltRPs) in the response to Zn^2+^ limitation. Furthermore, this study compares, corroborates and contrasts the findings from a similar study in Zn^2+^-limited *Mtb* (Dow et al., 2021). Zn^2+^-limited mycobacteria (i.e., *Mtb* and *Msm*) upregulate key antioxidant enzymes (*e*.*g*., catalase-peroxidase, alkylhydroperoxidase and thioredoxins) and many genes involved in homologous recombination and DNA repair. Both species also downregulate fatty acid biosynthesis and oxidative phosphorylation during Zn^2+^ limitation; mechanisms that are presumed to conserve reducing power (NADPH) and decrease endogenous oxidant production (Dow et al., 2021). These congruencies signify the importance of the oxidative stress response during Zn^2+^-limiting conditions in both pathogenic and non-pathogenic mycobacteria, possibly implicating its importance in other bacteria. Moreover, we demonstrate that the upregulated oxidative stress response in Zn^2+^-limited conditions does not depend on the expression of AltRPs in *Msm*. Finally, we describe the processes that are affected by expression of AltRPs during Zn^2+^-limitation, further signifying the role of these Zn^2+^-independent ribosomal protein paralogs in mycobacterial physiology (Figure 5).

The oxidative stress response is a conserved feature in Zn^2+^-limited mycobacteria and closely resembles the transcriptomic response to mycobacteria treated with oxidizing agents (Voskuil et al., 2011; Li et al., 2015). Mycobacteria exhibit two DNA damage response pathways; the LexA/RecA-dependent “SOS response” and a LexA/RecA-independent pathway (Müller et al., 2018). From this study, we conclude Zn^2+^ limitation likely induces the LexA/RecA-independent pathway of DNA repair, considering 83% of genes in the PafBC regulon (Müller et al., 2018) are upregulated in Zn^2+^-limited *Msm*. Because the SOS response is associated with bacterial persistence (Podlesek and Žgur Bertok, 2020), this finding has important implications for the role of Zn^2+^-limitation in mycobacterial physiology. Beyond the conserved response, *Mtb* and *Msm* exhibited many unique regulatory strategies for orthologous genes when facing Zn^2+^ limitation. Notably, *Mtb* downregulates key processes relevant to infection (*e*.*g*., *mce1C-F* and *mtrA*). Interestingly, decreased expression levels of these genes correlate with a hypervirulent phenotype (Shimono et al., 2003; Fol et al., 2006), and are in agreement with the increased virulence observed in our previous mouse infection study of Zn^2+^-limited *Mtb* (Dow et al., 2021). On the other hand, Zn^2+^-limited *Msm* specifically downregulates genes binding ATP and GTP and amino acid biosynthesis, while upregulating processes involved in redox homeostasis and repairing oxidized proteins and damaged DNA (beyond the DNA repair genes also upregulated in *Mtb*). Accordingly, the fast-growing *Msm* appears to have a more robust oxidative stress response in ZLM compared to its slow-growing relative. This observation could be explained by the different growth rates of these mycobacteria, especially considering that in the same media (i.e., ZLM), Zn^2+^-limited *Msm* has much lower cell density compared to the Zn^2+^-replete condition (Dow and Prisic, 2018), but Zn^2+^ supplementation has no effect on growth of *Mtb* (Dow et al., 2021). Moreover, Zn^2+^-limited *Msm* attains a considerably higher cell density than *Mtb* (in stationary phase of the respective growth curves in ZLM) (Dow and Prisic, 2018; Dow et al., 2021). Therefore, due to the increased biomass and biological demand for Zn^2+^, *Msm* likely experiences more severe Zn^2+^ limitation than *Mtb* in the same growth condition (i.e., ZLM). Logically, this extends to *Msm* enduring more severe oxidative stress and is a likely explanation for the more robust oxidative stress response observed in *Msm*.

This analysis of Zn^2+^-limited *Msm* expands the mechanisms relevant to combating oxidative stress that were previously described in *Mtb* (Dow et al., 2021), while highlighting divergent features of these mycobacteria when facing Zn^2+^-limitation. One such example is the differential regulation of ergothioneine biosynthesis, which was upregulated in *Msm*, but downregulated in *Mtb* during Zn^2+^ limitation. Ergothioneine is believed to function as an antioxidant, although its deletion from *Msm* did not affect survival upon exposure to oxidizing agents (Sao Emani et al., 2013). In addition, its biosynthesis in *Mtb* appears to be dictated by nutrient starvation, not oxidative stress (Richard-Greenblatt et al., 2015). Although we hypothesize that *Msm* experiences more severe oxidative stress in ZLM, thus providing credence to the specific induction of ergothioneine in *Msm*, this observation could, again, point to the presumed difference in nutrient demands for *Msm* and *Mtb* grown in ZLM. Another divergent example is the unique upregulation of pyrimidine and arginine biosynthesis in Zn^2+^-limited *Mtb*. Arginine limitation triggers oxidative damage and sterilizes *Mtb* (Tiwari et al., 2018), and our results suggest this might be a weakness unique to *Mtb*, since arginine biosynthesis was, in contrast, downregulated in Zn^2+^-limited *Msm* and therefore likely does not serve the same protective role during oxidative stress in *Msm* as it does in *Mtb*. Finally, Zn^2+^ limitation differentially altered the expression of certain transcription factors in mycobacteria, with the gene encoding WhiB6 induced in *Mtb* and the gene encoding WhiB7 induced in *Msm*. Both transcription factors have been implicated in maintaining redox balance (Burian et al., 2012; Chawla et al., 2012); however, WhiB6 also links to virulence in *Mtb* (Chen et al., 2016) and WhiB7 links to iron limitation in *Msm* (Geiman et al., 2006). The selective induction of different *whiB*-like genes shown here suggests mycobacteria have adapted altered usage of specific regulatory networks in response to limited Zn^2+^. In sum, while both mycobacteria upregulated strategies to combat oxidative stress upon Zn^2+^ limitation, there is evidence that *Mtb* has also evolved to use Zn^2+^ specifically as a cue in the context of infection.

Both mycobacteria altered carbon metabolism in response to Zn^2+^ limitation. However, they did so in unique ways, albeit presumably, to the same end. While Zn^2+^-limited *Msm* downregulated genes in the non-oxidative PPP and the downstream glycolytic enzymes *gap* and *pgk*, Zn^2+^-limited *Mtb* upregulated key enzymes in the oxidative PPP directly, i.e., glucose-6-phosphate dehydrogenase (*g6pd*) and 6-phosphogluconate dehydrogenase (*6pgd*), while downregulating phosphofructokinase-1 (*pfkA*), the first committed step in glycolysis (Dow et al., 2021). Both of these strategies allow glycolytic flux to be diverted through the oxidative PPP arm, which increases NADPH production to fuel cellular antioxidant systems (Mullarky and Cantley, 2015). Presumably, these divergent mechanisms work to achieve the same goal of increased NADPH production in Zn^2+^-limited mycobacteria, which is required to reduce thioredoxin and maintain antioxidant reducing power (Wong et al., 2017). The different routes taken to redirect glycolytic flux to combat oxidative stress (Mullarky and Cantley, 2015) could again be reflective of different growth characteristics. *Msm*, having a greater need to reduce cellular carbon for biomass, cannot afford an overall decrease in glycolytic flux and so depends on downregulating the non-oxidative PPP arm and subsequent glycolytic enzymes. *Mtb* on the other hand, could take a more direct approach to increasing flux through the oxidative PPP arm directly, by upregulating these enzymes and creating a bottleneck at commitment to glycolysis. These strategies reflect unique mechanisms to divert glycolysis for increased reducing power during oxidative stress via metabolic reprogramming in mycobacteria.

To further elucidate the role of AltRPs in the Zn^2+^-dependent response in *Msm*, we first investigated the concordance between gene expression patterns at the level of the transcriptome and proteome since AltRPs are implicated in conferring selectivity in the process of translation (Chen et al., 2020). We found that, regardless of AltRP expression, most proteins follow the expression pattern of the transcript. Notable exceptions involve protease systems. Although significantly upregulated at the transcript level in both wild type and Δ*altRP*, the ClpP1 peptidase component of the Clp protease (MSMEG_4673) (Akopian et al., 2012) was only upregulated at the protein level in Δ*altRP*. Furthermore, components the ClpB-DnaK bi-chaperone system, which reactivates aggregated proteins (Yin et al., 2021), showed opposing discordant regulation. ClpB subunit (MSMEG_0732) was upregulated at the transcript level only in wild type and upregulated at the protein level only in Δ*altRP*. Beyond that, although not differentially expressed at the transcript level in ether wild type or Δ*altRP*, DnaJ (a cochaperone that chauffeurs clients to the DnaK system) (Fay and Glickman, 2014) was downregulated at the protein level in wild type only. While our results do not necessarily implicate the direct involvement of AltRPs in the discordant regulation of these protease systems, they do indicate that AltRP expression may help preserve the fidelity of translation during Zn^2+^-limiting conditions, since there was an overabundance of the ClpP protease and protein delivery to the bi-chaperone system in the Δ*altRP* background. Underrepresented protein expression of translation initiation factor (IF-2) in AltRP-expressing cells suggests that one possible mechanism by which Alt-ribosomes may contribute to protein fidelity is by decreasing the rate of translation, perhaps to enable more efficient nascent protein folding. Indeed Alt-ribosomes are shown to exhibit a relative initiation defect and are suggested to be “slow and accurate” compared to primary ribosomes (Chen et al., 2020). This assumption is further supported by the increased abundance of trigger factor protein (MSMEG_4674), a ribosome associated chaperone, in the Δ*altRP* mutant.

By directly comparing the transcriptomes and proteomes of the Δ*altRP* mutant and the wild type, we were able to describe several processes that were differentially regulated upon the expression of AltRPs in the context of Zn^2+^-limitation (Figure 5). Remarkably, the Δ*altRP* mutant utilized the glyoxylate shunt via induction of isocitrate lyase (MSMEG_0911) while concurrently upregulating succinate dehydrogenase, an enzyme required in both the TCA cycle and the electron transport chain. During iron limitation, due to decreased activity of NADH dehydrogenase (containing seven Fe-S clusters), induction of the glyoxylate shunt may help rearrange bacterial metabolism to increase the efficiency of electron flow (Koedooder et al., 2018). Interestingly, turnover of Fe-S proteins appears to be elevated in the absence of AltRPs, since only the Δ*altRP* mutant upregulated the SUF operon at the protein level. This observation suggests that AltRPs may play a role in iron homeostasis during Zn^2+^-limiting conditions, which is consistent with the iron-limiting phenotype observed in the Δ*altRP* mutant (Chen et al., 2020). Accordingly, it’s tempting to speculate that the glyoxylate shunt is linked to iron-limiting conditions in the Δ*altRP* mutant, and by extension, that AltRP expression helps maintain iron homeostasis during Zn^2+^ limitation in mycobacteria. It is important to note, however, that the glyoxylate shunt is one of a few specific features of the Δ*altRP* mutant in *Msm* that mirrors the phenotype of Zn^2+^-limited (but AltRP-expressing) *Mtb.* Other overlapping phenotypes include downregulation of the arginine repressor (*argR*), the gene encoding the two-component response regulator DevR, and upregulation of key enzymes in the ox-PPP. These observations suggest the possibly that AltRPs have evolved to have unique roles in different mycobacterial species and highlights the need to decipher the exact role of AltRPs in pathogenic and non-pathogenic (or slow- and fast-growing) mycobacteria.

To that end, this multi-omics analysis shed light onto possible mechanisms that could be involved in the AltRP-dependent phenotype observed in Zn^2+^-limited *Msm*, specifically with regards to the elongated cell length phenotype (Figure 5). Certain processes involved in cell division were only differentially regulated during Zn^2+^ limitation when AltRPs were expressed. This includes the chromosomal replication initiation factor (*dnaA*) and the septum formation components (*sepF* and *pbpB*), which were downregulated during Zn^2+^ limitation, but not in the Δ*altRP* mutant. Further, the Δ*altRP* mutant upregulated expression of the gene encoding the transcriptional regulator WhmD (MSMEG_1831) compared to the wild type and complement strains in ZLM. WhmD has a role in septum formation and cell division, and decreased expression leads to elongated cells with diminished septum formation (Gomez and Bishai, 2000). Furthermore, two cell wall hydrolases, amidase AmiB (MSMEG_1679) and hydrolase (MSMEG_0117), were among the topmost upregulated genes in Δ*altRP* compared to the wild type and complement strains. Peptidoglycan hydrolases open the mesh-like peptidoglycan cell wall for insertion of new material (Kieser and Rubin, 2014), and AmiB is responsible for cell separation in *E. coli* (Uehara et al., 2010). Consistently, AmiB is strongly downregulated only in elongated cells (i.e., Zn^2+^-limited, AltRP-expressing cells). There were numerous processes involved in cell division that are uniquely regulated in the Δ*altRP* mutant compared to the other Zn^2+^-limited strains that could explain the failure of the Δ*altRP* mutant to elongate upon Zn^2+^ limitation.

This study demonstrates the benefit of the model mycobacterium, *Msm*, in evaluating the contribution of AltRPs in bacterial physiology, due the role of AltRP expression in the Zn^2+^-limited phenotype in *Msm*. Using this system, we were able to monitor changes in expression patterns of numerous genes and proteins that correlate with the phenotypic observations associated with AltRP expression in the context of Zn^2+^ limitation. It was intriguing that most of the differential regulation between the wild type and the Δ*altRP* mutant was at the level of the transcriptome, and it is not clear how AltRPs would regulate the expression of these genes. Interestingly, the expression levels of many transcriptional regulators were altered by the presence or absence of AltRPs, offering a potential explanation for the different transcriptional responses observed (Figure 5). These changes could stem from differences in post-transcriptional or post-translational processes in Zn^2+^-limited cells containing Alt- or Prim- ribosomes (i.e., wild type and Δ*altRP*, respectively), as a direct effect of selective translation, AltRP-specific interactions with other cellular components, or other unknown mechanisms. Indeed, deciphering mechanisms of AltRP-dependent gene regulation is a notable pursuit. All in all, we conclude that AltRPs are not only important as functional replacements for their Zn^2+^-dependent paralogues; they are also involved in the transcriptomic response to the Zn^2+^-limited environment.

## Data Availability Statement

The original contributions presented in the study are publicly available. This data can be found here: Transcriptomics data (RNA-seq) are available from the GEO database (GSE188233) and proteomics data are available from the PRIDE database (PXD029588).

## Author Contributions

AD and SP designed and conceptualized the study and edited the final manuscript. AD performed all experiments, did the bioinformatics analysis, and wrote the original manuscript. AB obtained tagwise counts from raw RNA-seq data. EM obtained spectral counts from raw proteomics data. All authors contributed to the article and approved the submitted version.

## Conflict of Interest

The authors declare that the research was conducted in the absence of any commercial or financial relationships that could be construed as a potential conflict of interest.

## Publisher’s Note

All claims expressed in this article are solely those of the authors and do not necessarily represent those of their affiliated organizations, or those of the publisher, the editors and the reviewers. Any product that may be evaluated in this article, or claim that may be made by its manufacturer, is not guaranteed or endorsed by the publisher.
