# Supplementary material for: Multi-Omics Profiling Specifies Involvement of Alternative Ribosomal Proteins in Response to Zinc Limitation in Mycobacterium smegmatis
Source: Front Microbiol. 2022 Feb 10;13:811774. doi: 10.3389/fmicb.2022.811774 (PMC8866557; doi:10.3389/fmicb.2022.811774)
Supplement: Supplementary file 16 [file Image_1.PDF]

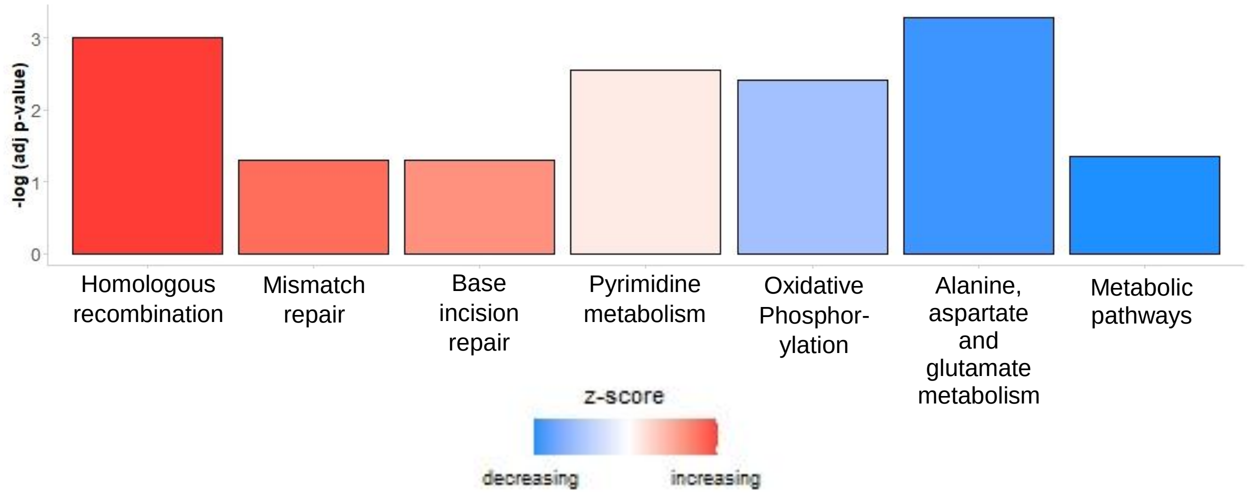

S1 Figure. KEGG terms enriched in the list of DE genes in Zn^{2+}-limited *Msm* (wild type ZLM vs. wild type ZRM). The enrichment analysis was conducted using DAVID. The z-score represents the likelihood that a given process is upregulated (red) or downregulated (blue) in the dataset and the analysis is described in Materials and Methods.
