# Supplementary material for: Multi-Omics Profiling Specifies Involvement of Alternative Ribosomal Proteins in Response to Zinc Limitation in Mycobacterium smegmatis
Source: Front Microbiol. 2022 Feb 10;13:811774. doi: 10.3389/fmicb.2022.811774 (PMC8866557; doi:10.3389/fmicb.2022.811774)
Supplement: Supplementary file 17 [file Image_2.PDF]

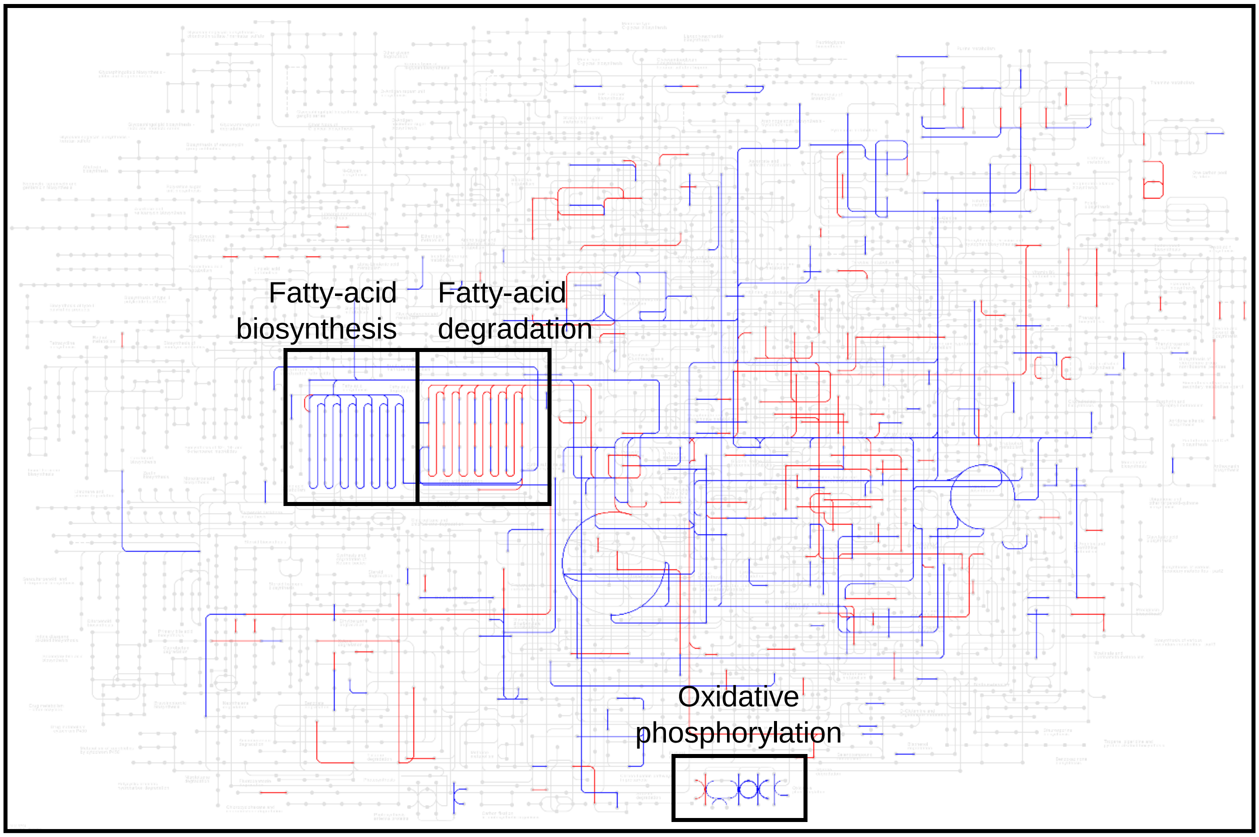

S2 Figure. DE genes in KEGG term ‘msm01100 metabolic pathways’ that are upregulated (red) and downregulated (blue) in Zn^{2+}-limited *Msm* (*i.e.*, ZLM vs. ZRM) are superimposed onto a global metabolic network. Enzymes (genes) are represented as lines and their products or substrates are represented by nodes. Only DE genes are colored, and relevant pathways are labeled on the map for clarity.
