# Supplementary material for: Multi-Omics Profiling Specifies Involvement of Alternative Ribosomal Proteins in Response to Zinc Limitation in Mycobacterium smegmatis
Source: Front Microbiol. 2022 Feb 10;13:811774. doi: 10.3389/fmicb.2022.811774 (PMC8866557; doi:10.3389/fmicb.2022.811774)
Supplement: Supplementary file 18 [file Image_3.PDF]

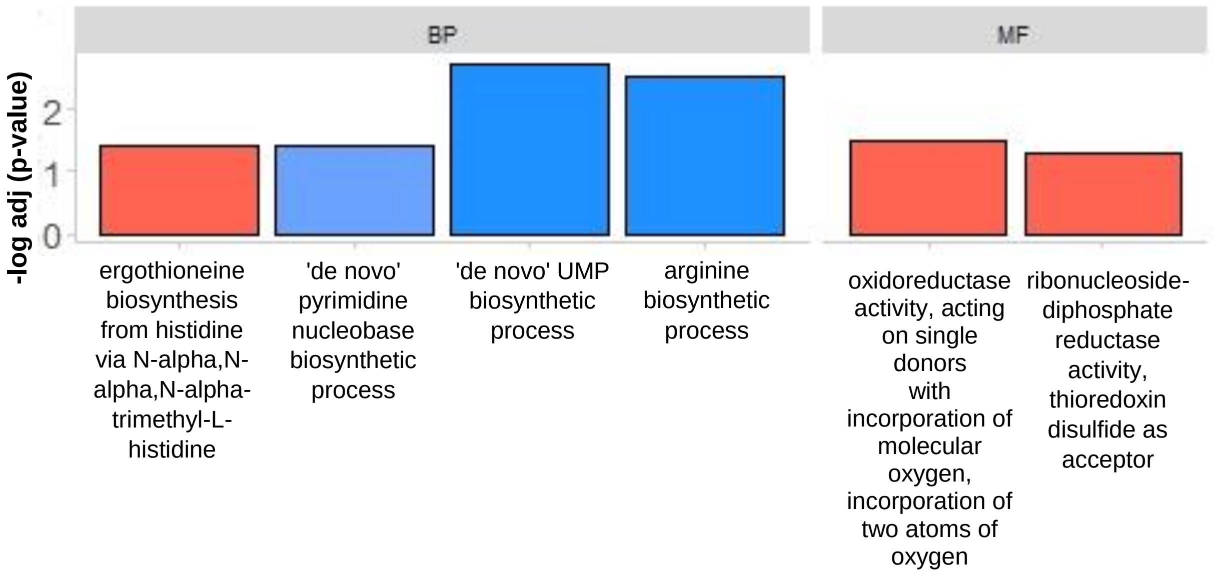

S3 Figure. GO terms enriched in the orthologous genes that are discordantly regulated in *Msm* and *Mtb*. The enrichment analysis was conducted using DAVID and MSMEG locus tags. The color of the bars represents whether the process is upregulated (red) in or downregulated (blue) in *Msm* vs. *Mtb*. The enriched GO terms are defined as ‘Biological Processes’ (BP) or ‘Molecular Functions’ (MF) as indicated at the top of the graph.
