# Supplementary material for: Multi-Omics Profiling Specifies Involvement of Alternative Ribosomal Proteins in Response to Zinc Limitation in Mycobacterium smegmatis
Source: Front Microbiol. 2022 Feb 10;13:811774. doi: 10.3389/fmicb.2022.811774 (PMC8866557; doi:10.3389/fmicb.2022.811774)
Supplement: Supplementary file 19 [file Image_4.PDF]

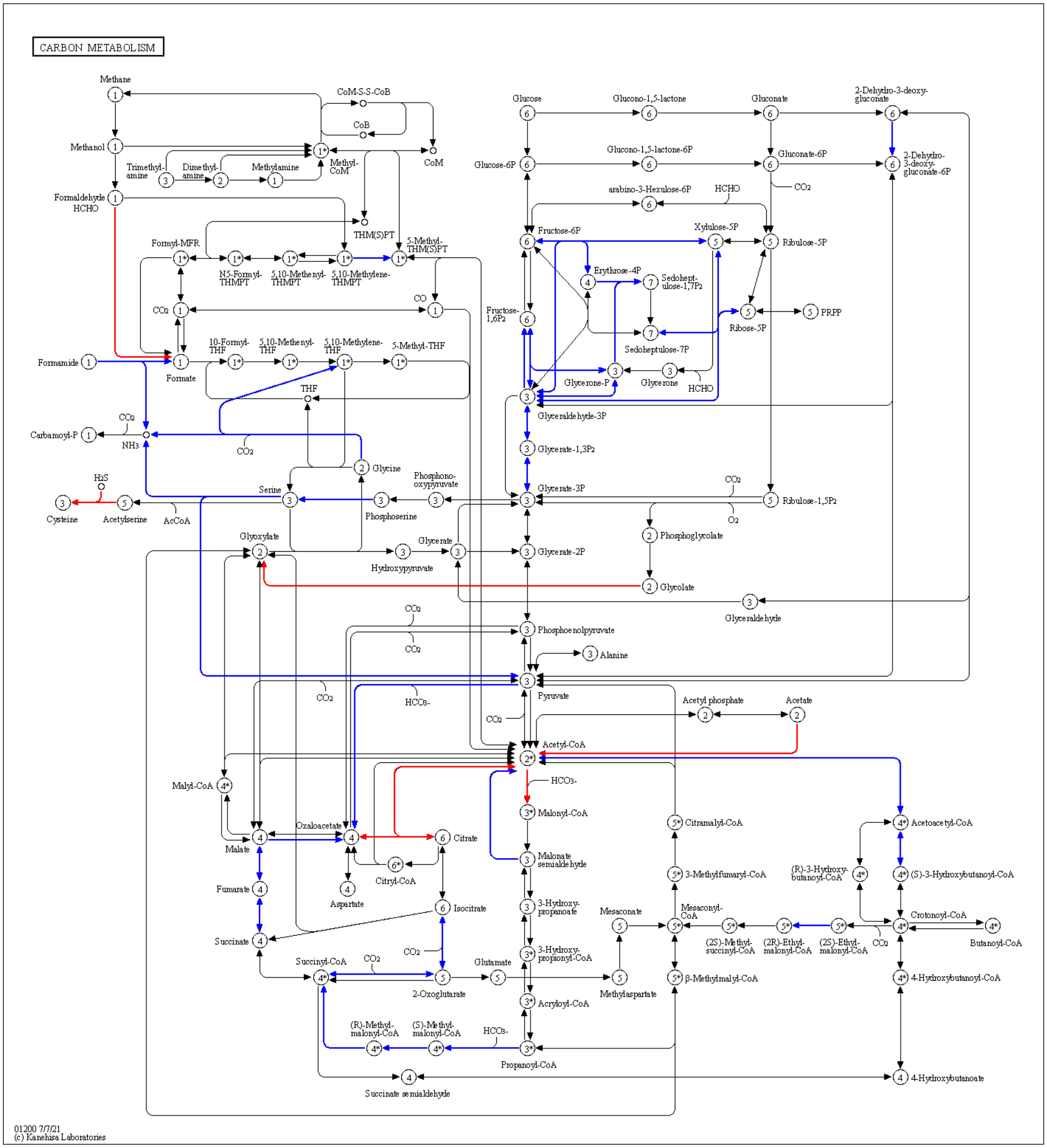

S4 Figure. DE genes in Zn^{2+}-limited *Msm* (*i.e.*, ZLM vs. ZRM) that are involved carbon metabolism as defined by KEGG term 'msm01200' are colored in red (upregulated) and blue (downregulated).
