# Supplementary material for: Multi-Omics Profiling Specifies Involvement of Alternative Ribosomal Proteins in Response to Zinc Limitation in Mycobacterium smegmatis
Source: Front Microbiol. 2022 Feb 10;13:811774. doi: 10.3389/fmicb.2022.811774 (PMC8866557; doi:10.3389/fmicb.2022.811774)
Supplement: Supplementary file 20 [file Image_5.PDF]

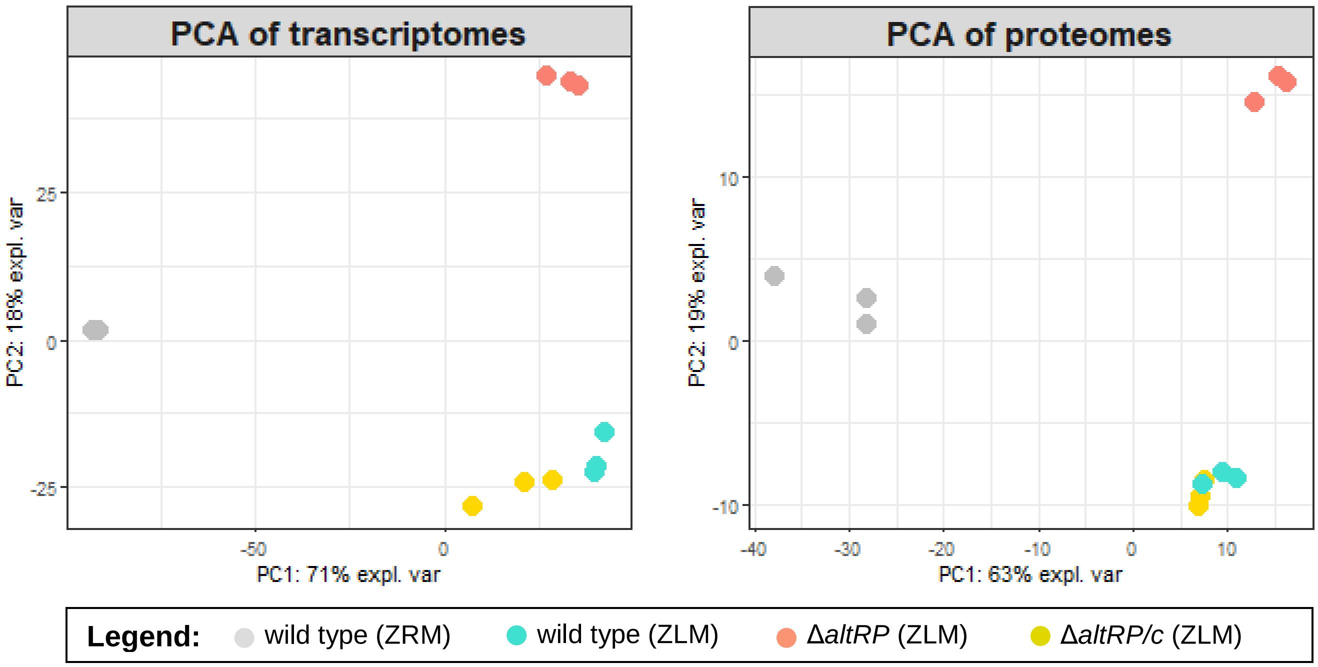

S5 Figure. Principal component analyses (PCA) of transcriptomes and proteomes from wild type *Msm* grown in ZRM along with the wild type, Δ*altRP* mutant and Δ*altRP/c* grown in ZLM. The axes of the PCA plots show the leading principal component (PC1) and the second principal component (PC2) driving variation in the dataset. The percentage of variation for each component is given in the axis label for each plot. The transcriptomes and proteomes were detected from the same biological replicates for each condition (n=3).
